# Highland and Islands Medical Service Board

**Published:** 1916-06-24

**Authors:** 


### HIGHLANDS AND ISLANDS MEDICAL SERVICE BOARD.

### A Policy of Cautiousness.

The first report of this Board was summarised in the Hospital of July 24, 1915, and the second report, bringing an account of the work accomplished during the year 1915, has just been issued. The purpose of the Board, stated shortly, is to secure an improved medical service for the poorer inhabitants of the Highlands and Islands of Scotland. To this end there is allotted by Parliament an annual sum of £42,000, and of this sum £6,700 was spent during the year on medical service, and nearly £1,000 on nursing service. It is a condition of the Parliamentary grant that any balance unexpended is not liable to surrender at the close of the financial year, and from the figures just quoted it is obvious that in the meantime this balance must amount to a relatively considerable sum. On December 31, 1915, the figures were nearly £68,000, to which there has to be added £42,000, being the Parliamentary grant for the current year. There are good and sufficient reasons for this accumulation of funds. First is the fact that it has only been possible hitherto to get a part of the scheme into operation. Information had to be gathered over a wide area and in districts where official investigations are conducted only with difficulty. Detailed negotiations with medical practitioners, with nursing organisations, with the Insurance Commissioners, and with various other bodies were imperative. In the very nature of things such matters take time, and this particularly when they deal with widely scattered districts where travelling facilities exist on but a limited scale. Then there has been the disturbing influence of the war, which has taken many from the usual scenes of their labours, both doctors and nurses. Proceeding from the same source, too, is the pressure of the Treasury, which has plainly intimated the desire of "Their Lordships" that expenditure shall be curtailed in all possible directions, even at the cost of the temporary postponement of some of the improvements contemplated by the Board. Still further, it has to be remembered that certain of these improvements will involve considerable capital expenditure, and this alone is sufficient to justify the Board in husbanding its resources. The times are not propitious for such expenditure. Labour is scarce and building materials are dear, and local authorities are not inclined for the costs of new enterprises. Hence additional hospital accommodation, and improved houses for doctors, and development of ambulance services, and extension of telegraphic and telephone facilities must be postponed to a happier day. Then an accumulated balance will have its opportunity, and the present policy of the Board its justification. Too enterprising a development cannot for the moment find either a financial or an ethical defence. The future will make large demands, and it would be an unfortunate impression to leave on local authorities that the Medical Service Board is a kind of fairy godmother who will do everything that is necessary. The Board will gladly give help to those who show reasonable readiness to help themselves, but it has no apparatus for the relief of those who have no effort of their own to offer. Those who are disposed to complain that the Board is hoarding public funds instead of spending them, forget the demands of the future and the necessity of giving aid in a fashion which shall not be demoralising to the recipients. On the question of money it would be ungracious not to remember that no remuneration is attached to membership of the Board.

### The Cottar's Plight.

The particular piece of work which the Board has accomplished is the organisation of arrangements by which persons of the crofter and cottar classes, and others in similar circumstances, may obtain medical help at fixed moderate fees, these fees being the same whatever the distance of the patient from the doctor's place of residence. At an early stage of the inquiry it became manifest that there was a vast amount of illness among poor non-pauper people, and that in consequence of the distances to be travelled the cost of medical advice often meant that such persons either did not send for a doctor at all or sent for him only when the position was beyond redemption. To those who live with omnibuses and taxicabs at every corner it is difficult to picture medical practice where the distances between patient and doctor are sometimes as high as fifty miles and distances of twenty-five to forty miles are not uncommon. As an example a case is quoted in which a doctor had to traverse ten miles by hill path in visiting a patient forty miles from his house, and fourteen miles by a similar path in attending another patient thirty-four miles away. No wonder they have time for thought in the Highlands! To meet such positions the Board has subsidised medical practitioners who accept the principle above defined, and medical practitioners are now working under the scheme in nearly all the districts which come within the area of the Board's operations. The financial and other advantages to the patients are obvious, but it remains to be seen whether the arrangements are fair to members of the medical profession. At the outset there was some anxiety and even controversy on this point. In the meantime the scheme is receiving a fair trial, and this particular issue will be settled by experience and by the working of the law of supply and demand. Practice in these remote and often isolated districts can at the best attract but a limited number of men, and unless the conditions are tolerable this number will certainly diminish. The Board is naturally anxious to utilise to the best advantage the funds at its disposal, and it is, of course, obvious that hospitals, nurses, ambulances, motor-cycles, telegraphs, and telephones are all of no avail unless behind the machinery there is the directing hand of the competent medical practitioner.

